# A Study of the Relationship Between Breastfeeding, Attachment Style and Oral Health in Pubertal Children: A Network Analysis

**DOI:** 10.3390/children13030421

**Published:** 2026-03-19

**Authors:** Jaime Alberto Toledo-Junco, Antonia Barranca-Enríquez, Tania Romo-González, Laura Leticia Salazar-Preciado, Clío Chávez-Palencia, Israel Huesca-Domínguez, Yolanda Campos-Uscanga, Socorro Herrera-Meza

**Affiliations:** 1 Laboratorio de Biología y Salud Integral, Instituto de Investigaciones Biológicas, Universidad Veracruzana, Xalapa 91190, Veracruz, Mexico; zs22024899@estudiantes.uv.mx (J.A.T.-J.); abarranca@uv.mx (A.B.-E.); ishuesca@uv.mx (I.H.-D.); 2 Centro Universitario de Tonalá, Universidad de Guadalajara, Tonalá 45425, Jalisco, Mexico; leticia.salazar@academicos.udg.mx (L.L.S.-P.); clio.chavez@academicos.udg.mx (C.C.-P.); 3 Instituto de Nutrición Humana, Universidad de Guadalajara, Guadalajara 44100, Jalisco, Mexico; 4 Instituto de Salud Pública, Universidad Veracruzana, Xalapa 91190, Veracruz, Mexico; ycampos@uv.mx; 5 Instituto de Investigaciones Psicológicas, Universidad Veracruzana, Xalapa 91190, Veracruz, Mexico; soherrera@uv.mx

**Keywords:** emotional bonding, exclusively breastfed, oral hygiene, caries, dental occlusion

## Abstract

**Highlights:**

**What are the main findings?**

• Exclusively breastfed children had less history of cavities, better oral hygiene, better occlusion, and had higher scores on the secure attachment style scale compared to those who were not exclusively breastfed.
• Breastfeeding may be associated with better health and development in the children sampled in the municipality of Azueta, Veracruz.

**What are the implications of the main findings?**

• Secure and closeness attachment can be considered a potential psychosocial pathway, linking early breastfeeding experiences with subsequent oral health patterns in middle childhood. That is, attachment styles seem to be multidimensionally integrated by biological-evolutionary, cognitive, behavioral, affective, and social aspects.
• It is important to study these phenomena from a comprehensive perspective, as this can guide public policies and the development of comprehensive health promotion programs.

**Abstract:**

**Background/Objectives**: Although the benefits of breastfeeding on the development and health of the infant are well known, the relationship between breastfeeding, oral health and attachment style or emotional bonding is not fully known. This research sought to explore, from a comprehensive perspective, the associations between breastfeeding history and children’s attachment styles, as well as the relationships between breastfeeding history and oral health indicators within conceptual psychophysiological frameworks discussed in the literature. **Methods**: This was a cross-sectional (descriptive and analytical) and correlational study. In this work, the associations of breastfeeding with attachment and oral health were analyzed in 100 children between 9 and 11 years old at a primary school in the municipality of José Azueta, Veracruz, Mexico, from December 2023 to September 2024 by a clinical history, dental examinations (Oral Hygiene Index-Simulated (OHI-S), Dental Caries History (DEOPT) and Detection of Malocclusions (DAI)) and the Attachment and Interaction Styles Instrument. **Results**: Significant differences were found in the security and closeness attachment style, the oral-hygiene index, the caries index, and occlusion by type of breastfeeding, showing better values in boys and girls who were exclusively breastfed. Likewise, both in the correlation analysis and in the multiple regression model, associations were observed between having been exclusively breastfed and the attachment style and oral indices. **Conclusions**: Our data show the importance of breastfeeding in pubertal children, since it was associated with better attachment and oral health; however, these findings reflect patterns of co-occurrence and should not be interpreted as causal relationships.

## 1. Introduction

Human milk is the natural and ideal food for newborn and nursing children due to its nutritional characteristics that contribute to harmonious growth throughout the life of the child. The previously mentioned is true if it is administered as the only food and nutrient on demand during the first six months of life and, after this age, it is complemented with adequate, timely, and safe foods, as suggested by the World Health Organization (WHO) and the United Nations Children’s Fund (UNICEF) [1].

In this sense, breastfeeding has been considered a natural process that aims to promote better nutritional and psychomotor development [2,3,4,5], as well as strengthen the bond between mother and child, which contributes to the emotional well-being of the infant. Therefore, breastfeeding has been related to the secure and closeness attachment style [6,7]. Bowlby, in his attachment theory, describes it as a tendency to establish intimate emotional ties with individuals, determined as a basic component of human nature present in an embryonic form in the newborn and continuing throughout life [6]. Based on this theory, three types or styles of attachments were described: (1) security and closeness attachment, (2) dependence and insecurity attachment, and (3) anxiety–avoidance attachment [8]. Childhood and puberty are crucial for the establishment of attachment styles, as this is when individuals learn to regulate their own behavior to promote security and thus are able to construct more flexible internal models. Considering that these styles can manifest as internal or external to the individual, one way to bring the styles together is to link the internality–externality dimension in interaction with that of attachment styles.

Furthermore, attachment style has been linked to psychological and biological systems that regulate threat assessment, stress response, and recovery. That is, stressful situations are thought to activate the attachment system, and physiological systems are an important mechanism for the expression of stress responses, such as activation of the hypothalamic–pituitary–adrenal (HPA) axis [9]. Likewise, attachment style and breastfeeding are referred to as two processes that are interrelated and that stimulate each other to achieve a relationship between mother and child [10]. From the perspective of attachment theory, frequent physical contact, mutual eye contact, on-demand comforting, and synchrony between mother and child during breastfeeding create conditions conducive to the formation of a secure and closeness attachment [11]. In recent years, these have gained importance since a secure and closeness attachment style favors the mother–child emotional ties and results in a better quality of breastfeeding, while promoting psychomotor development and optimal health for the infant.

In oral health, breastfeeding may also contribute indirectly through its association with secure and closeness attachment. That is, since breastfeeding has been shown to be significantly associated with secure and closeness attachment and less attachment disorganization [7], breastfed children are less prone to chronic stress. Since chronic stress or allostatic load can generate an increase in both saliva and its characteristics (i.e., protein concentration, cortisol levels and pH), leading to general pathologies in the mouth, it may result in a higher prevalence of dental caries and inflammatory processes in soft tissues [12,13,14,15]. In children, dental caries is considered one of the most notable and prevalent problems during their temporary dentition and in the development of mixed dentition due to the aforementioned, as well as considerable neglect by parents [16,17]. In that respect, human milk adapts to the nutritional and developmental needs of the infant. This is because it provides triglycerides and fatty acids and performs structural functions of the cell membrane, as well as providing minerals, such as calcium and phosphorus, and proteins, such as casein, that lead, among other things, to the remineralization of the enamel surface of the teeth. In addition, human breastmilk has several glycoproteins that could block the adhesion of Streptococcus mutans, a major cariogenic bacterial species, on tooth surfaces. It should be noted that the pH of human milk ranges between 7.1 and 7.7 and does not have any modifying effect on oral pH. That is, the buffering capacity of breastmilk could help limit caries formation and allow remineralization of non-cavitated lesions [18,19].

Although there are some studies that show the benefits of breastfeeding in the prevention of cavities, there are also others that reveal the relationship of breastfeeding with this condition [20,21]. For instance, several studies in children have shown that breastfeeding is associated with the presence and severity of caries, especially when breastfeeding is carried out for more than 6 to 12 months. Although they suggest that the presence of caries could be a consequence not only of prolonged breastfeeding, but also of a lack of oral hygiene with fluoride toothpaste, related to whether parents intervene in the application of these measures [16,17,22,23]. Furthermore, Shrestha et al. [24] warn against unrestricted nighttime breastfeeding after the eruption of the first primary tooth, arguing that nighttime feeding may increase the risk of caries in early childhood. However, as previously mentioned, there are also studies that propose that breastfeeding, in children who were breastfed until six months, together with good oral-hygiene habits, may be a protective factor against early-childhood caries [21,25,26,27]. An increased risk of childhood caries can be observed only when breastfeeding lasted up to 12 months. Lower risks of childhood dental caries were also found in infants who drank some breast milk (from the breast or with a bottle) compared to those who were never breastfed [24,27]. Likewise, when examining skulls of children from the Smithsonian Natural History Museum in Washington dated to prehistoric times, Palmer [28] showed that the vast majority of the deciduous teeth studied were free of caries, since, at that time, the only infant nutrition was breastfeeding, always for a prolonged period.

Moreover, the breastfeeding process promotes the development of the initial muscular action mechanisms governed by reflex arcs, which contribute to the establishment of proper occlusion [29,30,31]. This is because the breastfeeding process from an orthopedic point of view favors the first physiological advance of the child’s occlusion, which leads to the exercise of the masticatory muscles and stability between the mandible and the maxilla, allowing swallowing and, therefore, correct nasal breathing that could be associated with certain habits that put the stomatognathic system at risk [18,20]. In addition, suction activity promotes mandibular protrusion and retrusion movements, with simultaneous lingual movements that achieve swallowing and exert enough force to process food [31,32,33]. Nonetheless, evidence suggests that prolonged breastfeeding may negatively influence occlusion, as a persistent infant swallowing pattern caused by prolonged breastfeeding can lead to malocclusion and developmental disorders in both hard and soft tissues. The position of the infant’s mandible during prolonged breastfeeding is usually retruded due to the fact that the tongue is pressed between the dental arches (infant swallowing pattern). This positioning negatively influences muscle tension, favoring the muscles that retract the mandible, which becomes the basis for the development of a functional Class II malocclusion [13]. All this is of utmost importance since dental malocclusion is considered a public health problem in the pediatric population because it ranks third in oral health pathologies [34].

Despite growing evidence on the nutritional, immunological, and emotional benefits of breastfeeding [19,35], most studies have analyzed these dimensions in isolation. There is a notable lack of an integrative approach, examining how breastfeeding may simultaneously shape emotional development, particularly attachment patterns, and physiological outcomes such as oral health. This omission is especially relevant given that early feeding practices constitute one of the first embodied relational experiences between the infant and the caregiver [11], with potential long-term effects on both psychosocial and biological health outcomes. Moreover, the association between breastfeeding and caries is not only one of the most debated risk factors, but this uncertainty has also led medical and dental organizations to offer different recommendations [24]; thus, further study is warranted. By addressing this underexplored aspect, our research seeks to provide new insights into how early feeding behaviors shape the physical and psychological development of the child’s oral cavity and facial structures.

This gap is addressed by analyzing the plausibility of a shared psychobiological pathway through which breastfeeding may be related to emotional bonding but also to optimal oral function. Thus, this research sought to explore, from a comprehensive perspective, the associations between breastfeeding history and children’s attachment styles, as well as the relationships between breastfeeding history and oral health indicators within conceptual psychophysiological frameworks discussed in the literature. The goal is to guide the development of comprehensive health promotion programs and public policies that support breastfeeding.

## 2. Materials and Methods

### 2.1. Population and Sample

This was a cross-sectional (descriptive and analytical) and correlational study. It was conducted in José Azueta, Mexico, which is one of the 212 municipalities of the state of Veracruz de Ignacio de la Llave. It is located in the southern region of the state, known as the Papaloapan basin. The total population of this municipality is 2653 inhabitants. The human development index for this population is at the medium level (0.6704), where 82% know how to read and write, and the average educational level is primary school [36]. According to INEGI data, the state of Veracruz is ranked 15th in exclusive breastfeeding with a percentage of 29.5%, below the national average and well below the 2030 Sustainable Development Goals target of 70% [37,38].

The sample consisted of boys and girls who had or had not been exclusively breastfed. The following selection criteria was taken into account: (i) children between 9 and 11 years old belonging to the municipality of José Azueta, Veracruz, who were enrolled at the Leona Vicario school, whose guardians had authorized their participation through informed consent, and who had authorized their participation through informed assent; (ii) children who could not read and write or who found it difficult to complete any of the assessments were excluded; and (iii) those participants unable complete the instruments used or the oral assessment were also eliminated.

The sample size and the selection of participants were based on non-probabilistic convenience sampling. This was because studies on these topics are scarce, particularly regarding the relationship between all variables (oral health, breastfeeding, and attachment). Therefore, the study adopts an exploratory approach; moreover, the results are applicable only to the selected sample and to populations with similar characteristics [39].

The research protocol was approved by the Research Ethics Committee of the Institute of Psychological Research at Universidad Veracruzana, with registration number 202310, and the Research Committee of the Institute of Biological Research, with registration number 23-06. The information obtained was treated in a strictly confidential manner in accordance with the provisions of the Regulations of the Mexican General Health Law on Research. The clinical history (18 questions) was obtained from the children’s mothers or guardians. All dental examinations were conducted at the same time of day (to avoid saliva variation) and performed by a dental surgeon (author of this article) trained and calibrated to evaluate all indices. Examinations were performed in a classroom with an artificial light, mirror and explorer, millimeter ruler, and a periodontal probe. The Attachment and Interaction Styles Instrument (with 87 Likert-type-scale items), developed and validated by Rolando Diaz Loving and Ariadna Vargas González in primary school children between 9 and 13 years of age [40], was administered to the children after the oral assessment was completed. In this exercise, children freely choose their answers without any kind of pressure or manipulation. The child was supported with a ruler so they could follow the questions correctly and avoid confusion due to the number of questions in the instrument. Similarly, any questions they failed to comprehend were addressed to improve their understanding. The participants (children and their guardians) were provided with the results of the oral health assessments and information on the children’s attachment styles. Furthermore, when relevant data were found regarding oral health indices and attachment styles, the guardian was informed personally and referred for prompt dental or psychological care, as appropriate.

### 2.2. Instruments or Techniques for Data Collection

#### 2.2.1. General Health History

The clinical history (18 questions) was applied to the mothers. It included demographic data, information on feeding during the first six months of life and during the first year of age, hygiene habits, oral habits, and habits that can alter occlusion. In addition to being part of the diagnosis, these variables helped to avoid some confounding factors. To collect data on initial feeding history, mothers were asked whether their child had been exclusively breastfed or not, and they were given explanations of what these processes entail. They were also asked if they had used any aids for breastfeeding, and if so, they were asked to specify which ones. Finally, they were asked how long they had been breastfeeding, with the options being “Less than 6 months”, “6 months”, or “More than 6 months”. It is noteworthy that the meaning of each question was always explained to ensure accurate responses and avoid any interference due to lack of information [Clinical Record-Supplementary Materials].

The type of feeding was classified as “exclusively breastfed” (if the infant received exclusively human milk); and “not exclusively breastfed” (if the infant received mixed breastfeeding—partial human milk or human milk substitutes). That is, according to the Mexican Standard, exclusive breastfeeding (EBF) is defined as feeding infants with human milk as their sole source of nutrition. In addition, they may only receive oral rehydration solution, drops or syrups containing vitamin or mineral supplements, or medications [41].

#### 2.2.2. Simplified Oral Hygiene Index (OHI-S)

A dental index that allows quantitative evaluation of the degree of oral hygiene (determination of tartar or dental calculus on teeth) was applied. The dental examinations were performed by only one dental surgeon trained and calibrated to evaluate this index in order to avoid intra- and inter-examiner error (with kappa values greater than 0.80) for dental assessment. The evaluation included six dental surfaces on primary and permanent teeth (mixed dentition); thus, the maxillary and mandibular arches are divided into sextants, each of which will be represented by a surface: (1) the vestibular surfaces of an upper central incisor, (2) lower central incisor, and (3) upper right and (4) left first molar; on lingual surfaces the (5) lower right and (6) left first molars were also assessed. With a microbrush and gentian violet or, if applicable, a plaque-revealing tablet, the dental surface was painted to assess violet stains and the degree of deposits. If no staining was found on the vestibular surface of the tooth, it was classified as 0; stains on one-third of the surface were recorded as 1; two-thirds as 2; and finally, category 3 was assigned if the entire crown was stained. Subsequently, all the values obtained for each subject were added and their arithmetic mean was taken. Thus, the sum of the results for all surfaces was divided by 6 in each individual [42]. If the mean ranged between 0 and ≤0.9, it was considered optimal, from 1.0 to ≤1.9 regular, from 2.0 to ≤2.9 bad, and values over 3.0 were considered very bad.

#### 2.2.3. DEOPT Index

The DEOPT index is the sum of carious primary and permanent teeth (mixed dentition), with indication for extraction and filling; please note that missing teeth were not considered in this index. The indicated extraction is defined as necessary when a pathology does not respond to the most frequently used treatment; restoration by means of a crown is considered a filled tooth; when the same tooth was filled and decayed, the most serious diagnosis was recorded. Moreover, the presence of sealants was not quantified.

This index is used to assess the history of caries in a child with deciduous dentition. Thus, we used the letter D for decayed teeth, E means extracted due to caries, and the letter O means filled or restored teeth. The 20 deciduous dentition teeth were evaluated (in the case of finding permanent teeth, teeth 16, 26, 36 and 46 were included, among others). If any tooth was missing, we verified the cause, for instance, if it was due to anodontia, etc. Supernumerary teeth were not considered for this study. Caries history with the DEOPT index can be assessed in two different ways: (1) individually, as the results from the sum of all the teeth with caries alteration in each subject, and (2) communally, as the average result of all the participants. It is noteworthy that all dental surfaces must be dry to carry out this index. The dental examinations were performed by only one dental surgeon trained and calibrated to evaluate this index in order to avoid intra- and inter-examiner error (with kappa values greater than 0.80) for dental assessment [43].

#### 2.2.4. Dental Aesthetic Index (DAI)

This index has been widely used and recommended by the WHO, which establishes a list of occlusal features or conditions in categories, ordered on a scale of degrees that allows observing the severity of malocclusions. It has been widely reproduced as it guides the assessment based on orthodontic treatment needs in the population under study. In addition, its use in epidemiological and public health applications is well recognized, as it is a reliable and valid tool [44] for both adult and pediatric populations [45]. The DAI is given by a standard regression equation composed of 10 occlusal characteristics, with their corresponding regression coefficients, with exact and rounded values. These components must be measured, and their values are then multiplied by a corresponding coefficient for each component. The DAI value is obtained by adding these results, plus a constant of 13. The DAI has allowed public dental health and orthodontic insurance programs in different countries to select and identify people eligible for these types of programs based on their aesthetic needs. Since it does not require a specialist, it can be carried out with limited resources and has been effective when managing public funds [46]. However, for this study, a trained dentist was employed; this individual’s approach had been previously standardized and calibrated by orthodontic specialists to ensure that the application of the DAI was as accurate as possible. Finally, it is important to highlight that, given the developmental stage of the studied children, the diagnosis reported through the DAI must be viewed as an initial resource that should be followed up on, considering possible changes for non-nutritive sucking habits as well as their treatment [47].

#### 2.2.5. Attachment and Interaction Styles Instrument for Boys and Girls

We used the instrument for measuring attachment styles, developed by Rolando Diaz Loving and Ariadna Vargas González, in primary school children between 9 and 13 years of age. It is made up of 87 Likert-type-scale items, with response options of 1 = never; 2 = rarely; 3 = sometimes yes, sometimes no; 4 = often; and 5 = always [40]. As mentioned earlier, this instrument is divided into 7 factors; within the security and closeness attachment style, there is factor 2: external security with an Alpha of 0.8368; factor 3: internal security with an Alpha of 0.6569; and factor 7: interdependence–closeness–expressiveness with an Alpha of 0.7781. For the dependency and insecurity attachment style, we find factor 5: worried–friendly with an Alpha of 0.7292; and factor 6: anxious–dependent with an Alpha of 0.7033. Finally, there is the anxiety and avoidance attachment style that corresponds to factor 1: avoidant–anxious–aggressive with an Alpha of 0.7778; and factor 4: avoidant–independent with 0.7111 [40]. This instrument was designed and validated in the Mexican population, thus being a viable, reliable and culturally sensitive contribution for obtaining data on attachment styles in boys and girls during this school stage. Additionally, the instrument was piloted prior to its application, and the examiner was trained to administer it. Likewise, for this study, the Alpha values were calculated for each attachment factor as follows: factor 1 = 0.71, factor 2 = 0.84, factor 3 = 0.67, factor 4 = 0.58, factor 5 = 0.82, factor 6 = 0.70 and factor 7 = 0.71.

### 2.3. Data Analysis

Descriptive statistics such as frequencies, proportions, medians and interquartile ranges (IQRs) were performed to explore oral health conditions (oral indices, oral habits and oral hygiene) and attachment styles as a function of breastfeeding and sex. In the absence of normality and variance homogeneity in the data (Table S1—Supplementary Materials), the Mann–Whitney sum rank test and the Kruskal–Wallis tests were applied to compare between two or more groups, respectively. Spearman correlations were also calculated across the entire data set. Variables with correlations equal to or greater than 0.8 were excluded from subsequent analyses to avoid collinearity in the data. Initially, a multiple regression analysis was performed to identify the variables that best explain oral health conditions, using attachment styles, the presence/absence of exclusive breastfeeding, and sex as predictor (or independent) variables, and OHI-S, DAI, and DEOPT as dependent variables. The variables were transformed using a logarithmic function (x + 1) for the models because they did not show a normal distribution. In turn, the best regression model was identified based on the minimum value of the Akaike Information Criterion (AIC). In this regard, it is important to mention that the variables of oral habits and oral hygiene were not included in the multiple linear regression analyses due to the large number of response categories and the sample size. However, oral hygiene was considered in the OHI-S. All statistical analyses were carried out in R-project version 4.4.2 [48].

#### 2.3.1. Network Analysis

A network approach was used to examine and interpret the conditional independence relationships between the set of variables, allowing for the assessment of relationships between variables while controlling for the effects of all other variables.

The network approach provides a more intuitive interpretation of the dataset by illustrating how variables co-occur through their direct relationships, where each variable is represented as a node and the thickness of the links represents the intensity of the relationships between pairs of nodes [49]. A mixed graphical model (MGM) was used through the mgm R package [50] to construct the network, since it allows integrating continuous (variables related to attachment and oral health) and categorical variables (variables related to demographics and breastfeeding). To estimate the network structure, a value of k = 2 was used to specify pairwise interactions in the model. The regularization parameter was selected using 10-fold cross-validation, which produces less-conservative models than the Extended Bayesian Information Criterion (EBIC), while balancing complexity and predictive power, facilitating the identification of interpretable associations. In turn, the “OR” rule was used to identify the importance of the links within the network, which highlights those links that are shown at least once in the regression models performed in the analysis. These criteria allowed the identification of relevant and interpretable relationships within the network. The graphical representation of the network was performed using the qgraph R package [51].

#### 2.3.2. Centrality Analysis

The importance of each node within the network can be quantified by centrality measures [52]. These were calculated using the qgraph R package [51]. The centrality values were presented as standardized values (z-score). As a measure of centrality, expected influence was calculated, defined as the sum of the weights of all edges connected to a node. Unlike other centrality measures, this metric retains the sign of the associations, considering both positive and negative relationships, which allows a more accurate evaluation of a node’s influence within the network.

#### 2.3.3. Network Stability

Model stability was assessed using the mgm R package [50], employing the resample function to generate 1000 empirical sampling distributions based on a non-parametric bootstrap. Subsequently, the plotRes function from the same package was used to visually represent the sampling distributions using the 5% and 95% quantiles. Stability is represented as the percentage of bootstrap samples in which each edge was estimated to be non-zero, with higher percentages indicating greater stability of the results (Figure S2—Supplementary Materials). Additionally, the correlation stability coefficient (CS) was estimated using non-parametric resampling with 1000 samples implemented in the bootnet R package [53]. In general, CS values > 0.25 indicate that the network analysis results show moderate stability, whereas values greater than 0.5 indicate strong stability.

## 3. Results

### 3.1. Description of Participants

A total of 120 children were invited to participate, and 100 (51% girls and 49% boys) provided informed consent. Of these, 52 were exclusively breastfed and 48 were partially breastfed. The children were in grades 4, 5, and 6 (ages 9 to 11) at the Leona Vicario Elementary School in the municipality of José Azueta, Veracruz, from December 2023 to September 2024. Of the 52% of children who were exclusively breastfed, 58% were girls and 42% were boys. Breastfeeding lasted 6 months for 63.33% of the girls who were exclusively breastfed. In contrast, breastfeeding lasted less than 6 months for 51.85% of the boys who were not exclusively breastfed (Table 1).

Habits, hygiene, and health status for teeth in the sampled children were variable. For instance, 63% responded that they had a daily frequency of 2–3 brushing instances, with girls who were not EBF having the highest percentage (71.43%). Regarding the time spent using a toothbrush, the group who were EBF reported that they had used their toothbrush for 0 to 3 months, with girls having the highest percentage (43.33%); whereas boys who were not EBF had the highest percentage of toothbrush use for over 7 months (48.14%). On the other hand, the group with lower percentages in bad oral habits was the EBF, since 83.30% of girls and 63.63% of boys lacked these traits. In addition, it should be noted that finger sucking was more frequent (28.57%) in girls and oral breathing was more common in boys (22.22%) who were not EBF. It is worth mentioning that only the duration of breastfeeding by gender was statistically significant when exclusive breastfeeding was present (*p* = 0.027) (Table 1).

### 3.2. Exclusive Breastfeeding (EBF) and Oral Health Status

**Figure 1:**
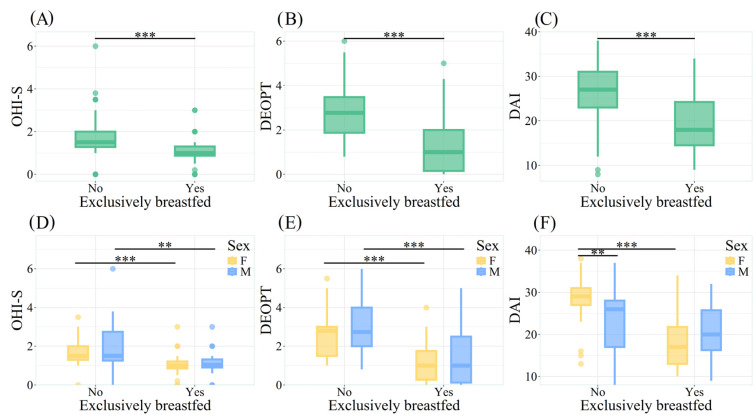
Oral Hygiene Index-Simulated (OHI-S), Dental Caries History (DEOPT) and Detection of Malocclusions Dental Aesthetics Index (DAI) in children (ages 9–11) with and without breastfeeding (N = 100). ** *p* < 0.01, *** *p* < 0.001. OHI-S: Oral Hygiene Index-Simulated; DEOPT: Decayed, Extracted, and Obturated Primary Teeth; DAI: Dental Aesthetic Index.

Oral hygiene (OHI-S) was significantly different according to food type consumption (*p* < 0.001). The group that was EBF and had “good” hygiene showed lower values (1.00 ± 0.43) than children who were not EBF and showed “regular” hygiene (1.50 ± 0.73) (Figure 1A, Tables S2 and S3—Supplementary Material).

Regarding the history of dental caries, children who were EBF had an average of 1.00 (±1.85), whereas children who were not EBF had an average of 2.77 (±1.60). This difference was significant (*p* < 0.001) (Figure 1B, Table S2 and S3—Supplementary Materials).

Detection of dental malocclusions (DAI) showed that EBF children fell within the “normal occlusion” range (18.00 ± 9.75), whereas non-EBF children were placed within the “defined malocclusion” range (27.00 ± 8.00). This difference was also significant (*p* < 0.001) (Figure 1C, Table S1—Supplementary Materials). Notably, girls had a higher contribution to these differences in both the EBF and non-EBF groups. Furthermore, within the non-EBF group, statistically significant differences were observed between girls and boys (*p* <.01), with the average being higher in girls, indicating worse occlusion (Figure 1F, Tables S2 and S3—Supplementary Materials). Additionally, the oral health variables (OHI-S, DEOPT, and DAI) also showed significant differences with respect to breastfeeding duration (*p* < 0.05), a variable derived from the clinical history (Table S4—Supplementary Materials).

### 3.3. Exclusive Breastfeeding (EBF) Attachment Styles

**Figure 2:**
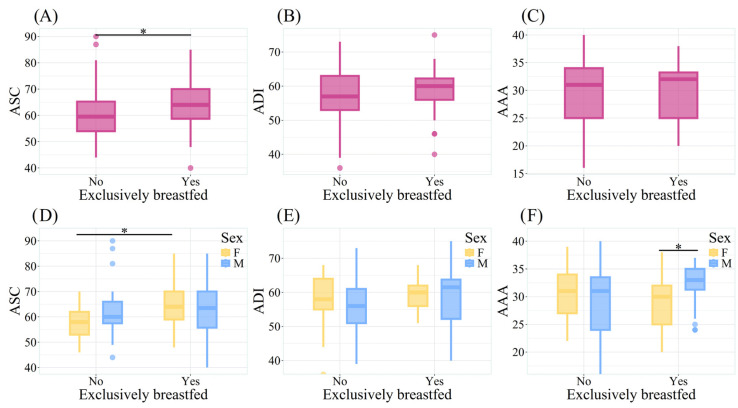
Attachment styles in boys and girls (ages 9–11) with and without exclusive breastfeeding (N = 100). * *p* < 0.05. A.S.C: Attachment style of security and closeness; A.D.I.: attachment style of dependence and insecurity; A.A.A: attachment style of anxiety and avoidance.

Differences in attachment style were significant (*p* = 0.036) for EBF (64.00 ± 11.25) vs. non-EBF children (59.50 ± 11.25) in security and closeness (Figure 2A). This difference was also driven by girls (Figure 2D). Similarly, the EBF group showed higher and statistically significant averages in factors 2, “external security”, and 3, “internal security”, which belong to the attachment style of security and closeness (Figure S1—Supplementary Materials). Regarding the attachment style of dependence and insecurity and the attachment style of anxiety and avoidance, no significant differences were found between the two groups (Figure 2B,C).

However, in the case of the attachment style of anxiety and avoidance, boys who were EBF had a higher and statistically significant score when compared to girls (Figure 2F), as well as for factor 1, “avoidant–anxious–aggressive”, of the dependency and insecurity style and factor 6, “anxious–dependent”, of the anxiety and avoidance style (Figure S1—Supplementary Materials).

### 3.4. Relationship Between Breastfeeding, Attachment Style and Oral Health

As shown in Table 2, the correlation analysis showed significant associations of attachment styles with oral health scores. The variables ASC, ADI, and AAA showed the highest correlations in the dataset (>0.8). In the case of the DEOPT, it showed negative associations with all of the following: “factor 2: external security” (−0.232); “factor 7: interdependence–closeness–expressiveness” (−0.217), which belongs to the attachment style of security and closeness; “attachment style of security and closeness” (−0.240); and “factor 5: worried–friendly” (−0.219), which belongs to the style of dependence and insecurity; meanwhile, it showed a positive association (0.383) with the OHI-S (Table 2).

Likewise, the Dental Aesthetic Index (DAI) showed a negative correlation with the “dependent and insecure attachment” (−0.204), and a positive correlation with the OHI-S (0.298) and the DEOPT (0.283). Malocclusion scores were positively associated with caries and oral hygiene indices, but also suggested that the dependent and insecure attachment could favor malocclusion either directly or perhaps because of the use of a finger, a pacifier, or some other bad habit. In addition, when analyzing the correlations by type of breastfeeding, it is interesting to note that several of the correlations for the oral indices with attachment are lost in children without EBF (OHI-S vs. F3 internal security, OHI-S vs. DEOPT, DEOPT vs. DAI) as well as between the attachment factors and styles (Table 2).

On the other hand, the multiple linear regression analysis to evaluate the relationship between breastfeeding, attachment, and sex and oral health (Table S5—Supplementary Materials) showed that breastfeeding has a significant association with OHI-S and DAI in the best-fitting models (Table 3); while DEOPT, in addition to showing an association with breastfeeding, also showed a significant relationship with duration of breastfeeding, and factor 7 (interdependence–closeness–expressiveness), which is part of the secure and close attachment style (Table 3).

**Figure 3:**
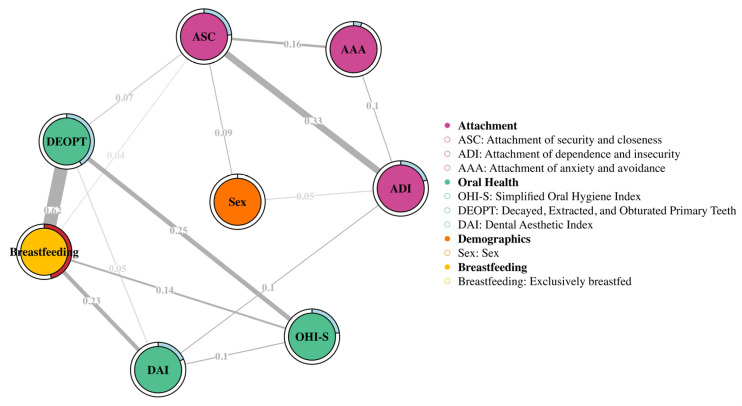
Network structure with variables of breastfeeding, oral health and attachment. The colors represent each construct being evaluated. The size of the edges represents the strength of the association in the network analysis. Gray edges indicate weights (coefficients) between nodes. Rings on nodes indicate R^2^ (continuous variable, light blue) or categorical accuracy (categorical variable, red).

Finally, based on the correlations between the studied variables (breastfeeding with attachment, oral health and gender), the network analysis showed the existing connections and their spatial distribution (Figure 3). Model stability was assessed with higher percentages indicating greater stability for the results, which support the assumed relationships (Figure S2—Supplementary Materials). Additionally, the correlation stability coefficient (CS) was 0.36, indicating moderate stability of the results. However, these relationships should be taken with caution. The network was estimated both for attachment factors (Figure S3—Supplementary Materials) and attachment styles. Here, the attachment styles are interpreted because they represent the overall pattern of the results (Figure 3). Edge values represent the weights of the associations, with thicker edges indicating stronger associations and thinner edges indicating weaker ones.

Consequently, when the connections and their distribution are analyzed, breastfeeding shows direct and significant relationships with the caries index, oral hygiene, and occlusion, being stronger with the caries index and occlusion (Weight = 0.62). It is worth noting that the association between the caries index (DEOPT) and breastfeeding, as well as factor 7 (interdependence–closeness–expressiveness), was significant in the multiple regression model. Regarding the associations between breastfeeding and attachment styles, only a moderate association was observed between breastfeeding and attachment style of security and closeness (ASC) (Weight = 0.04). For the associations between oral health indices and attachment styles, an association was only found between the caries index (DEOPT) and ACS (Weight = 0.07) and between the occlusion index (DAI) and attachment style of dependence and insecurity (ADI) (Weight = 0.1), although these were moderate associations.

Moreover, although in the previous analyses the sex led to significant differences only in the Dental Aesthetic Index and the attachment style of anxiety–avoidance, other differences were presented in the network analysis, although with moderate weights. For instance, sex was also associated with attachment style of security and closeness (Weight = 0.09) and with the attachment style of dependence and insecurity (Weight = 0.05) (Figure 3). Furthermore, the lack of association between breastfeeding and sex remained unchanged.

**Figure 4:**
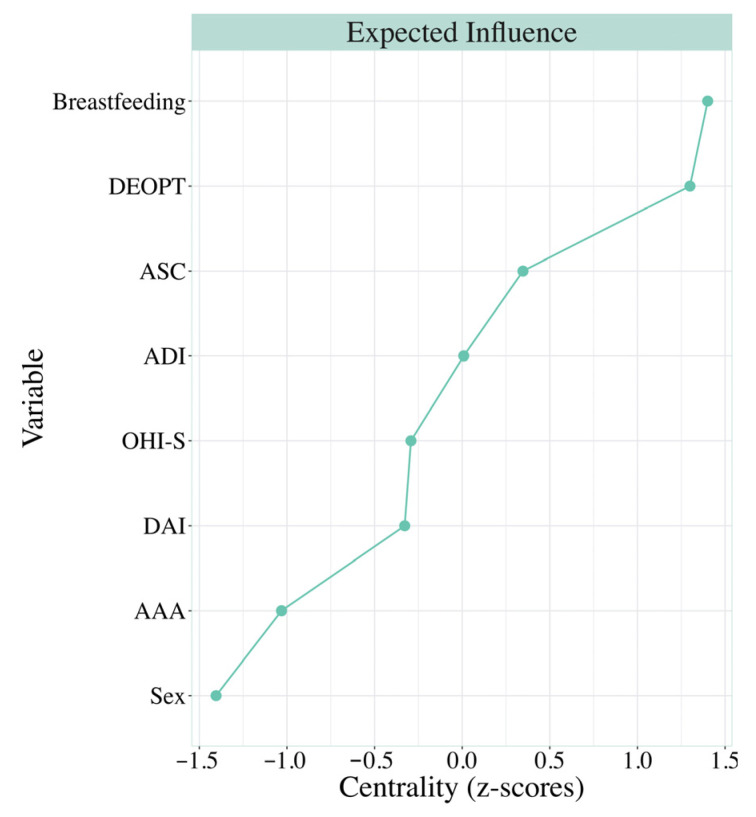
Standardized centrality of the strength of the variables breastfeeding, oral health, attachment style and sex of the estimated network. A.S.C: Attachment style of security and closeness; A.D.I.: Attachment style of dependence and insecurity; A.A.A: Attachment style of anxiety and avoidance; OHI-S: Oral Hygiene Index-Simulated; DEOPT: Decayed, Extracted, and Obturated Primary Teeth; DAI: Dental Aesthetic Index; Breastfeeding: exclusively breastfed.

On the other hand, when analyzing the centrality, we noted that nodes were not equally important (Figure 4). For example, among the categorical variables, breastfeeding was the node with the highest centrality in the network, whereas sex showed the lowest centrality value among all nodes. Regarding the oral health nodes, DEOPT showed higher centrality, while DAI showed lower centrality. Finally, among the attachment nodes, the security and closeness style showed higher centrality, whereas the anxiety and avoidance style showed lower centrality.

## 4. Discussion

Breastfeeding is a process that may be related to changes in the development of children across different life stages. However, despite the fact that scientific studies evidence the benefits it provides for the comprehensive health of human beings, some countries still concentrate the lowest percentages of exclusive breastfeeding (EBF). Among these is Mexico, where only 34.2% of children under six months of age receive EBF [54]. Although the American Academy of Pediatrics mentions that breastfeeding rates have increased significantly (i.e., from 2009 to 2018, there was an increase from 13.0% to 20.7%) [3], by 2019, 28.3% of children < 6 months received EBF; however, 42.9% of children < 12 months consumed infant formula, and only 29% continued to breastfeed at two years of age [55]. In addition, there are controversies about the influences of breastfeeding, not only for nutrition, but also on oral health and the emotional realm. Therefore, this research sought to explore the associations between breastfeeding history, attachment style and oral health from a comprehensive perspective.

Our results showed that children who were exclusively breastfed had significantly better oral hygiene than those who were not. Regarding the prevalence of dental caries, on average, children who were exclusively breastfed had better scores than children who were not EBF. This difference was statistically significant and indicates an association between breastfeeding history and dental caries indicators. These findings are consistent with previous studies showing a positive association of breastfeeding on the oral health of children, by contributing to healthy oral development and the low prevalence of oral pathologies, such as dental caries [25]. Tham et al. [56] mention that breastfeeding promotes the proper development of oral muscles, the formation of dental organs and less bacterial colonization in the mouth, all these factors being related to oral hygiene [57]. Moreover, differences observed in the oral hygiene and caries indices are discussed in relation to the previous literature on the composition of human milk, which, as an organic food, does not contain added sugars or artificial components that aid the proliferation and accumulation of bacteria, increasing chances of developing dental caries, as is the case with some formulas or already processed foods. Furthermore, human milk contains immunoglobulins, proteins, and enzymes that promote a healthier oral environment and therefore reduce the likelihood of caries formation [58]. Likewise, exclusive breastfeeding often delays the introduction of sugary foods, which are a known risk factor for tooth decay [59]. On the other hand, children who were not exclusively breastfed, when fed with formula or when other solid foods were introduced as complementary feeding, may have been more exposed to a higher risk of plaque formation and, in turn, the presence of tooth decay, due to the proliferation of cariogenic microorganisms. In this sense, Cui et al. [60] highlight that children fed exclusively with human milk until six months are less likely to develop tooth decay compared to those who receive formulas with added sugars.

On the other hand, the sucking process during breastfeeding has been linked to a more natural cleaning of the oral cavity, reducing the accumulation of bacterial plaque in the teeth [61,62]. This association of sucking, hygiene and the presence/absence of caries was also observed in the correlations for children with exclusive breastfeeding. Conversely, those who were not EBF showed no relation when the indices of caries, hygiene and dental aesthetics were analyzed. In addition, sucking was associated with dental malocclusions, since breastfed children presented normal occlusion, unlike children without EBF, who were in the range of “defined malocclusion”, and these differences were also statistically significant. These mechanical benefits of the breastfeeding process may involve the exercise of the masticatory muscles and the stability between the mandible and the maxilla (mandibular protrusion and retrusion movements) with simultaneous lingual movements allowing swallowing and, therefore, correct nasal breathing that could be associated with certain habits that put the stomatognathic system at risk [18,32]. On the other hand, the case of occlusion showed significant differences not only between dietary groups, but also by sex, especially in those individuals who did not have EBF, with girls having the highest average, that is, the worst occlusion. The latter may be attributed to the fact that girls with no EBF had a higher prevalence of finger sucking, unlike boys, who had more oral breathing. In this regard, Cedeño Romero and Vélez Lucas [63] suggest that the need to suck can be satisfied through nutritive sucking, including breastfeeding, and that when the need is not met, the child uses the finger, which creates a powerful vacuum inside the mouth. This vacuum applies forces to the teeth in the upper and lower jaws, causing the teeth to change their position over time. As the child grows, the aspiration present in digital sucking causes the palate to be pushed upwards and narrowed, leading to a misaligned bite. However, the better hygiene, lower rates of cavities, and fewer malocclusions found in children who were exclusively breastfed could also be explained by the mother–child bond that breastfeeding provides through skin-to-skin contact, which generates a secure and closeness attachment style in children. To test this hypothesis, the instrument validated for Mexican Children developed by Rolando Díaz Loving and Ariadna Vargas González [40] was used. This instrument was divided into seven factors: Within the security and closeness attachment style, there is factor 2: external security; factor 3: internal security; and factor 7: interdependence–closeness–expressiveness. For the dependence and insecurity attachment style, we find factor 5: worried–friendly, and factor 6: anxious–dependent. Finally, in the anxious and avoidant attachment style, there is factor 1: avoidant–anxious–aggressive; and factor 4: independent–avoidant [40]. In addition, the age range was established based on oral health, when children are in a mixed dentition process, which refers to the transition period from temporary to permanent dentition. In this stage (9 to 11 years old), childhood and puberty are crucial to establishing attachment styles and shaping them into adolescence and adulthood, being able to regulate their own behavior to promote safety, build more flexible internal models, differentiate more details, and also learn to convince others of their intentions [40]. Therefore, children begin to experience changes in their attitudes and begin to explore their environment.

In this regard, our results showed that children who were exclusively breastfed differed statistically, having higher security and closeness attachment style than those who were not, as reported by De Carmargo et al. [6]. In addition, the network analysis showed that breastfeeding was associated with oral aesthetics (DAI) and attachment style of security and closeness, and also DAI was associated with attachment style of dependence and insecurity, which could partly explain why the girls who were not exclusively breastfed had more thumb sucking and more malocclusions. This is consistent with the current understanding of child development, which suggests that sucking behaviors also arise and continue due to psychological needs [63].

Moreover, multiple regression analysis showed significant relationships between caries history and the attachment style of security and closeness through factor 7 (interdependence–closeness–expressiveness) and between the dental indices and breastfeeding. Additionally, breastfeeding, history of caries (DEOPT), and secure and close attachment showed important roles in the network centrality indices, whereas gender had very low centrality values. In addition, although the oral indices were not directly related to all the attachment styles, the caries index was associated with the security and closeness attachment style, and oral aesthetics (DAI) with the dependence and insecurity attachment style. These findings can be interpreted within theoretical frameworks linking attachment patterns and stress regulation, although causal pathways were not tested.

One possible mechanism involved in the association of these variables is the activation of the attachment system by stressful situations. Hence, breastfed children are expected to be less prone to chronic stress and, therefore, to inflammatory processes in soft tissues and caries formation, associated with a higher cariogenic bacterial load and with thinner and softer enamel surfaces, impaired dentin formation, and pulp chamber size [15]. In turn, this may also lead to a higher prevalence of dental caries and general pathologies in the mouth [12,13,14,15], while favoring unhealthy behaviors either in hygiene or by having higher consumption of sugars and fats in emotional eating [64]. Furthermore, since breastfeeding has been associated with greater maternal sensitivity in dyadic interactions [11], it is theoretically plausible that relational factors relate to caregiving behaviors; however, these mechanisms were not evaluated directly. Thus, secure and closeness attachment can be considered a potential psychosocial pathway, linking early breastfeeding experiences with subsequent oral health patterns in middle childhood. That is, attachment styles seem to be multidimensionally integrated by biological-evolutionary, cognitive, behavioral, affective, and social aspects [6].

Admittedly, our study is limited by its cross-sectional nature, which prevents establishing causal relationships. Also, memory biases (maternal recall bias) could be introduced when questioning mothers about breastfeeding that occurred years ago. In this regard, several studies have shown that this recall bias is multifaceted; that is, the accuracy of maternal recall can be affected by socioeconomic status, social norms, memory accuracy, or differential recall. Therefore, this bias involves not only remembering the infant’s feeding history but also reporting it truthfully. In this sense, Sorce et al. [65] propose not only monitoring recall accuracy but also asking about the certainty of the recall during interactions with healthcare providers, which provides an additional tool for assessing infant feeding. Furthermore, while the differences between reference and recall increase with longer recall periods (24 h to 20 years), given that recall of exclusive breastfeeding has been shown to be at least as accurate as recall of non-exclusive breastfeeding, recall questions can be particularly useful for monitoring the effects of interventions to improve exclusive breastfeeding [65,66].

Likewise, although some potential confounding factors that can influence caries and occlusion were incorporated, such as breastfeeding duration (which showed statistically significant differences between the breastfeeding groups and in the regression models with the caries index), hygiene and parafunctional habits (which did not show differences between the groups), the analysis did not incorporate other factors that are also important, such as exposure to fluoride (water, varnishes), diet or sugar consumption, bad habits, socioeconomic level, family health behaviors, maternal education, and previous access to dental care, among others [19]. Therefore, it is necessary to test the influence of these factors in future studies. Additionally, no questions were asked about the feeding techniques used by mothers during the first six months of life or whether children were breastfed at night. Moreover, despite having applied a regularization method to discard spurious associations in the network, the small sample size may increase the likelihood of overfitting, making it necessary to conduct further studies to support the results presented here and to generalize them to other populations. Finally, it is important to mention that the instrument assessed children’s attachment styles but not their mothers’. The absence of the latter prevents generating a more complete understanding of the associations regarding the dyad’s behavior. Hence, further data are desirable using standardized definitions regarding duration, frequency, and type of breastfeeding, as recently suggested by Shrestha et al. [24], as well as including other socioeconomic, demographic and behavioral factors [7,19,20,21].

## 5. Conclusions

Our results show that the group of exclusively breastfed children had less history of cavities, better oral hygiene, and better occlusion compared to those who were not exclusively breastfed. Furthermore, the exclusively breastfed children had higher scores on the secure attachment style scale. Thus, our findings show that breastfeeding may be associated with better health and development in the children sampled in the municipality of Azueta, Veracruz, but also support the need to study these phenomena from a comprehensive perspective. This can guide public policies and the development of comprehensive health promotion programs. While it is necessary to continue promoting breastfeeding, it is also essential to train health professionals such as general practitioners, nurses, and midwives to educate parents on healthy habits that include: oral hygiene practices, visits to an oral health professional by the child’s first birthday or within six months of the eruption of their first tooth, and behavioral modifications (such as minimizing nighttime feedings on demand and sleeping with the nipple in the mouth, cleaning gums after feeding, or offering water after feeding to rinse milk) to ensure optimal health outcomes that do not compromise the benefits of breastfeeding.

## Figures and Tables

**Table 1:** Frequencies and weighted percentages of population characteristics and habits.

			Exclusive Breastfeeding					Not Exclusively Breastfed ^a^				
Variable	Total (n = 100)		Girls (n = 30)		Boys (n = 22)			Girls (n = 21)		Boys (n = 27)		
	F	%	F	%	f	%	*p* ^b^	f	%	f	%	*p* ^b^
		**Duration** **of breastfeeding**										
Less than 6 months	30	30	2	6.67	5	22.73	0.027	9	**42.85**	**14**	**51.85**	0.634
6 months	33	33	**19**	**63.33**	6	27.27		3	14.48	5	18.52	
More than 6 months	37	37	9	30.00	**11**	**50.00**		9	42.85	8	29.63	
		**How many times do you brush your teeth?**										
0–1 times	37	37	11	36.66	8	36.36	0.999	6	28.57	12	25	
2–3 times	63	63	19	63.33	14	63.64		**15**	**71.43**	15	55.55	0.408
More than 3 times	0	0	0	0	0	0		0	0	0	0	
		**Toothbrush usage time**										
0–3 months	35	35	**13**	**43.33**	9	40.90	0.416	6	28.57	7	25.92	0.634
4–6 months	20	20	9	30.00	3	13.64		5	23.81	3	6.25	
7 months or more	34	34	6	20.00	7	31.82		8	38.10	**13**	**48.14**	
Does not remember	11	11	2	6.67	3	13.64		2	9.52	4	14.81	
		**Oral habits**										
Oral breathing	6	6	2	6.67	2	9.09	0.274	2	9.52	6	**22.22**	0.634
Finger sucking	15	15	1	3.33	4	18.18		6	**28.57**	4	14.81	
Nail biting	5	5	1	3.33	0	0		2	9.52	2	7.41	
Use of pacifier	9	9	1	3.33	2	9.09		1	4.76	5	18.52	
Atypical swallowing	0	0	0	0	0	0		0	0	0	0	
Biting objects	2	2	0	0	0	0		1	4.76	1	3.70	
None	57	57	25	**83.30**	14	63.63		9	42.86	9	33.33	

^a^ This group includes infants who were fed with formula, whether partially or exclusively; ^b^ Chi-square test excluding rows with zeros.

**Table 2:** Spearman correlation analysis in boys and girls.

	F1	F2	F3	F4	F5	F6	F7	A.S.C.	A.D.I.	A.A.A.	OHI-S	DEOPT
**Totals**												
**F2**	0.04											
**F3**	−0.16	0.76 ***										
**F4**	0.27 **	−0.20	−0.32 **									
**F5**	−0.08	0.51 ***	0.44 ***	0.00								
**F6**	0.20	−0.12	−0.13	0.12	−0.11							
**F7**	−0.02	0.63 ***	0.63 ***	−0.21 *	0.38 ***	0.07						
**ASC**	−0.05	0.91 ***	0.90 ***	−0.27 **	0.50 ***	−0.07	0.85 ***					
**ADI**	0.08	0.30 **	0.24 *	**0.09**	0.68 ***	0.65 ***	0.34 ***	0.33 ***				
**AAA**	0.77 **	−0.11	−0.39 **	0.82 ***	−0.05	0.20	−0.15	−0.21 *	0.11			
**OHI-S**	−0.00	−0.07	−0.08	−0.15	−0.04	0.01	0.06	−0.03	−0.02	−0.10		
**DEOPT**	0.01	**−0.23** *	−0.19	0.15	−0.22 *	−0.01	−0.22 *	−0.24 *	−0.17	0.10	0.38 ***	
**DAI**	0.08	−0.15	−0.18	0.03	−0.20	−0.07	−0.12	−0.17	−0.20 *	0.07	0.30 **	0.28 **
**With exclusive breastfeeding**												
**F2**	−0.02											
**F3**	−0.12	0.79 ***										
**F4**	0.29 *	−0.30 *	−0.41 **									
**F5**	−0.20	0.50 ***	0.44 **	−0.03								
**F6**	0.17	−0.17	−0.24	0.16	−0.22							
**F7**	0.09	0.66 ***	0.65 ***	−0.18	0.34 *	−0.11						
**ASC**	−0.05	0.91 ***	0.92 ***	−0.34 *	0.47 ***	−0.19	0.82 ***					
**ADI**	−0.01	0.31 *	0.20	0.06	0.61 ***	0.53 ***	0.33 *	0.29 *				
**AAA**	0.83 ***	−0.19	−0.33 *	0.72 ***	−0.13	0.20	−0.08	−0.24	0.05			
**OHI-S**	0.14	−0.18	−0.28 *	0.03	−0.19	−0.05	−0.20	−0.22	−0.239	0.14		
**DEOPT**	0.09	−0.15	−0.14	0.12	0.03	−0.06	−0.21	−0.15	−0.05	0.15	0.57 ***	
**DAI**	0.23	0.00	−0.10	−0.03	0.10	0.04	−0.02	−0.04	−0.06	0.18	0.39 **	0.35 *
**Not exclusively breastfed**												
**F2**	−0.02											
**F3**	−0.16	0.51 ***										
**F4**	0.41 **	0.02	−0.18									
**F5**	0.20	0.50 ***	0.38 **	0.09								
**F6**	0.35 *	0.03	−0.01	0.20	0.23							
**F7**	−0.12	0.49 ***	0.52 ***	−0.12	0.27	0.12						
**ASC**	−0.14	0.84 ***	0.78 ***	−0.08	0.46 **	0.07	0.80 ***					
**ADI**	0.31 *	0.23	0.15	0.19	0.66 ***	0.82 ***	0.19	0.24				
**AAA**	0.72 ***	−0.05	−0.24	0.90 ***	0.16	0.28	−0.16	−0.16	0.26			
**OHI-S**	−0.17	0.15	0.20	−0.17	0.07	0.04	0.23	0.22	0.09	−0.22		
**DEOPT**	0.03	−0.09	−0.03	0.17	−0.11	0.15	−0.16	−0.16	0.03	0.11	0.28	
**DAI**	0.02	−0.07	−0.01	−0.05	−0.19	−0.01	−0.19	−0.12	−0.09	−0.01	0.35 *	0.12

Note. High and/or significant values are bold. * *p* < 0.05, ** *p* < 0.01, *** *p* < 0.001. F1: Factor 1, avoidant–anxious–aggressive; F2: factor 2, external security; F3: factor 3, internal security; F4: factor 4, independent–avoidant; F5: factor 5, worried–friendly; F6: factor 6, anxious–dependent; F7: factor 7, interdependence–closeness–expressiveness. A.S.C: Attachment style of security and closeness; A.D.I.: attachment style of dependence and insecurity; A.A.A: attachment style of anxiety and avoidance; OHI-S: Oral Hygiene Index-Simulated; DEOPT: Decayed, Extracted, and Obturated Primary Teeth; DAI: Dental Aesthetic Index.

**Table 3:** Best predictor variables in multiple regression analysis using attachment styles, presence/absence of exclusive breastfeeding, and sex as predictors of oral health conditions.

Variable	SS	Df	F Value	*p*
DEOP (R^2^ = 0.40)				
Breastfeeding	1.074	1	31.831	**0.000**
Duration	0.393	2	5.821	**0.004**
F4	0.073	1	2.163	0.145
F7	0.218	1	6.456	**0.013**
OHI-S (R^2^ = 0.13)				
Breastfeeding	0.426	1	15.399	**0.000**
DAI (R^2^ = 0.14)				
Breastfeeding	0.284	1	13.472	**0.000**
F5	0.080	1	3.797	0.054

Note: High and/or significant values are bold. F4: Factor 4, independent–avoidant; F5: factor 5, worried–friendly; F7: factor 7, interdependence–closeness–expressiveness. DEOPT: Decayed, Extracted, and Obturated Primary Teeth; OHI-S: Oral Hygiene Index-Simulated; DAI: Dental Aesthetic Index; Duration: breastfeeding duration; Breastfeeding: exclusively breastfed; SS: Sum of Squares; Df: degrees of freedom; R^2^: coefficient of determination; *p*: significance.

## Data Availability

All data is available at https://github.com/ihuesca/DataBase_Breastfeeding/ (accessed on 31 January 2026).

## Supplementary Materials

The following supporting information can be downloaded at https://www.mdpi.com/article/10.3390/children13030421/s1: Clinical record; Figure S1: Attachment styles in boys and girls with and without exclusive breastfeeding; Figure S2: Bootstrap sampling distribution of edge weight estimates between pairs of nodes; Table S1: Normality test (Shapiro–Wilk) of the studied variables; Table S2: Frequencies and percentages of oral indices; Table S3: Medians, IQRs of the oral indices and the styles and factors of attachment by group (With exclusive breastfeeding and Not exclusively breastfed) and gender.
