# Supplementary material for: A Study of the Relationship Between Breastfeeding, Attachment Style and Oral Health in Pubertal Children: A Network Analysis
Source: Children (Basel). 2026 Mar 19;13(3):421. doi: 10.3390/children13030421 (PMC13025161; doi:10.3390/children13030421)
Supplement: Supplementary file 1 [file children-13-00421-s001.zip › children-4171455-supplementary.pdf]

CLINICAL RECORD

Date: ___________

Full Name: ______________________________________ Age: __________

Date of birth: ___________________________ Sex: _______________

Does your child have any illness? Yes ( ) No ( ) Which one?_______________________

Is your child taking any medication? Yes ( ) No ( ) Which one?______________________

HYGIENE HABITS

How many times does your child brush their teeth a day? 0-1 time ( ) 2-3 ( ) More than 3 ( )

Use oral hygiene aids: Toothpaste ( ) Dental floss ( ) Mouthwash ( )

How long has your child been using his or her current toothbrush? ____________________

How much time does your child spend brushing his teeth? ____________________

What do you consider to be the correct amount of time to use a toothbrush?

Less than 1 min ( ) 1 min ( ) 2 min or more ( )

OCCLUSION HABITS

Do you have any childhood habits that alter occlusion? Oral breathing ( ) Thumb sucking ()

Nail biting ( ) Pacifier use ( ) Atypical swallowing ( ) Biting objects ( ) None ( )

Do you have dental crowding? Yes ( ) No ( )

INITIAL FEEDING

Breastfeeding:

Exclusive: Do you feed your child exclusively with breast milk? Yes ( ) No ( )

Do I use any aids to drink milk? Yes ( ) No ( ) Which one? ___________________

How long did you breastfeed your child? Less than 6 months ( ) 6 months ( ) More than 6 months ( )

Supplemented: What other methods or foods do I use?

Table S1. Normality test (Shapiro–Wilk) of the studied variables

**Variable**	**W**	**p**
F1	0.972	0.031
F2	0.981	0.151
F3	0.951	< .001
F4	0.976	0.063
F5	0.893	< .001
F6	0.987	0.453
F7	0.96	0.004
ASC	0.966	0.01
ADI	0.977	0.082
AAA	0.962	0.006
OHI-S	0.883	< .001
DEOPT	0.928	< .001
DAI	0.955	0.002

Table S2. Frequencies and percentages of oral indices

			**Exclusive breastfeeding**				**Not exclusively breastfed**			
**Variable**	**Total (n=100)**		**Girls (n=30)**		**Boys (n=22)**		**Girls (n=21)**		**Boys (n=27)**	
	**f**	**%**	**f**	**%**	**f**	**%**	**f**	**%**	**f**	**%**
**OHI-S**										
Excellent	13	13	5	33.33	4	18.18	1	4.76	3	11.11
Good	33	33	17	46.66	7	31.82	6	28.57	3	11.11
Regular	49	49	8	36.67	11	50.00	13	61.90	17	62.96
Bad	5	5	0	0	0	0	1	4.76	4	14.81
**DEOPT**										
Very low	42	42	**22**	**42.30**	13	25	4	8.33	3	6.25
Low	22	22	5	9.61	4	7.69	5	10.41	8	16.66
Moderate	25	25	3	5.76	5	9.61	7	14.58	10	20.83
High	11	11	0	0	0	0	5	10.41	6	12.5
Very high	0	0	0	0	0	0	0	0	0	0
**DAI**										
Normal occlusión	60	60	**26**	**50**	16	30.76	7	14.58	11	22.91
Malocclusion defined	25	25	1	1.92	3	5.76	8	16.66	13	27.08
Severe malocclusion	14	14	3	5.76	3	5.76	6	12.5	2	4.16
Very severe malocclusion	1	1	0	0%	0	0	0	0	1	2.08

Table S3. Medians and IQRs of the oral indexes and the styles and factors of attachment by group (with exclusive breastfeeding and not exclusively breastfed) and gender

						Exclusive breastfeeding				Not exclusively breastfed				
	Exclusive breastfeeding		Not exclusively breastfed			Girls		Boys		Girls		Boys		
Variable	N	Median(IQR)	n	Median(IQR)	p	N	Median(IQR)	n	Median(IQR)	n	Median(IQR)	n	Median(IQR)	p
Attachment														
F1	52	11.00(4.25)	48	11.00(4.00)	0.431	30	10.00(3.75)	22	12.00(3.00)	21	10.00(10.0)	27	11.00(5.50)	0.048*
F2	52	22.00(5.00)	48	20.00(4.00)	0.02*	30	22.00(4.75)	22	22.50(6.00)	21	19.00(3.00)	27	21.00(4.00)	0.072
F3	52	25.50(4.250)	48	24.00(4.00)	0.017*	30	25.00(5.00)	22	26.00(4.75)	21	23.00(4.00)	27	24.00(4.50)	0.047*
F4	52	19.00(3.25)	48	19.50(5.25)	0.857	30	19.00(3.75)	22	19.00(3.00)	21	19.00(5.00)	27	20.00(5.00)	0.93
F5	52	26.00(5.00)	48	24.00(4.25)	0.118	30	26.00(3.00)	22	24.50(13.3)	21	24.00(4.00)	27	25.00(6.00)	0.102
F6	52	35.00(6.25)	48	32.00(7.50)	0.449	30	33.00(5.00)	22	37.00(6.50)	21	34.00(8.00)	27	32.00(6.50)	0.061
F7	52	16.50(3.00)	48	16.00(3.25)	0.583	30	17.00(2.00)	22	16.00(3.00)	21	15.00(3.00)	27	16.00(3.50)	0.188
ASC	52	64.00(11.25)	48	59.50(11.25)	0.036*	30	64.00 (11.0)	22	63.50(14.3)	21	58.00(9.00)	27	60.00(8.50)	0.057
ADI	52	60.00(6.25)	48	57.00(10.00)	0.239	30	60.00(6.00)	22	61.50(11.5)	21	58.00(9.00)	27	56.00(10.0)	0.512
AAA	52	32.00(8.25)	48	31.00(9.00)	0.706	30	30.00(7.00)	22	33.00(3.75)	21	31.00(7.00)	27	31.00(9.50)	0.091
Oral Health														
OHI-S	52	1.00(0.43)	48	1.50(0.73)	< .001*	30	1.00(0.37)	22	1.04(0.42)	21	1.50(0.70)	27	1.50(1.49)	< .001*
DEOPT	52	1.00(1.85)	48	2.77(1.60)	< .001*	30	1.00(1.48)	22	1.00(2.38)	21	2.80(1.50)	27	2.74(2.00)	< .001*
DAI	52	18.00(9.75)	48	27.00(8.00)	< .001*	30	17.00(8.75)	22	20.00(9.50)	21	29.00(4.00)	27	26.00(11.0)	< .001*

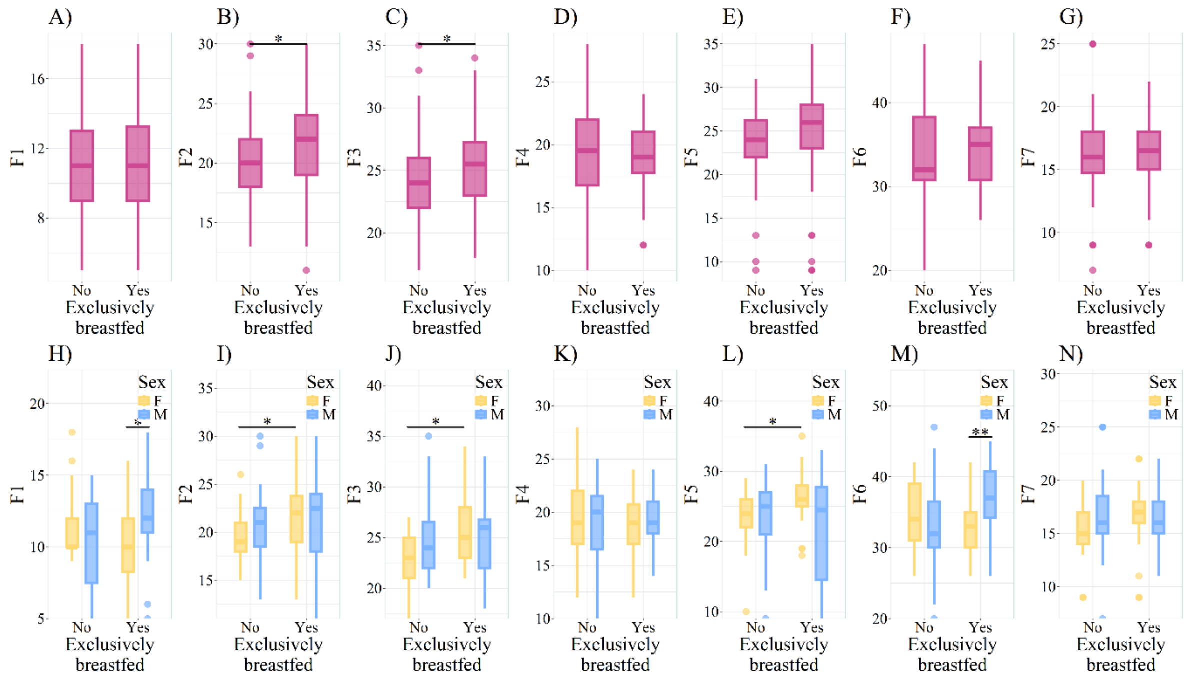

Figure S1. Attachment styles in boys and girls with and without exclusive breastfeeding. * p < .05, ** p < .01. F1: Factor 1, Avoidant–Anxious–Aggressive; F2: Factor 2, External Security: F3: Factor 3, Internal Security; F4: Factor 4, Independent–Avoidant; F5: Factor 5, Worried–Friendly; F6: Factor 6, Anxious–Dependent; F7: Factor 7, Interdependence–Closeness–Expressiveness.

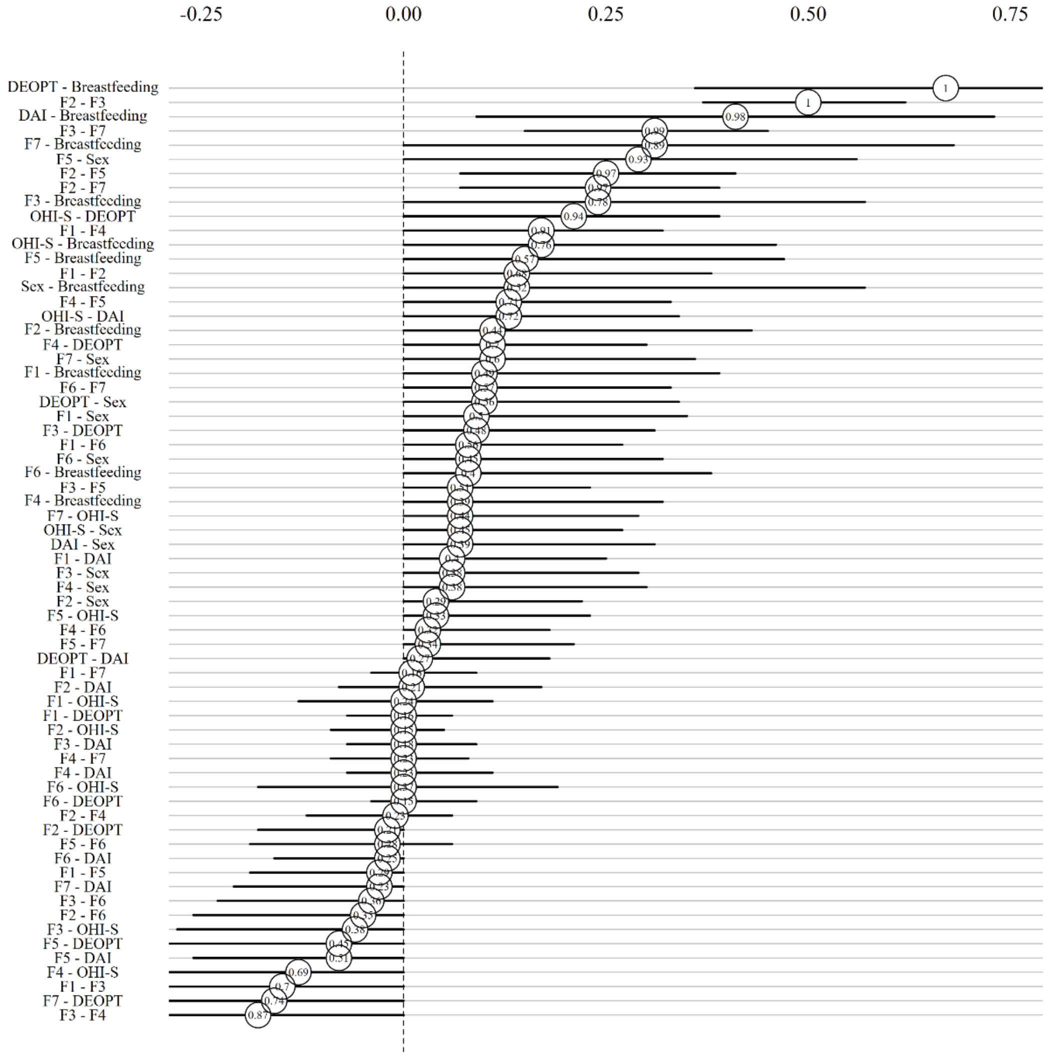

Figure S2. Bootstrap sampling distribution of edge weight estimates between pairs of nodes
